# Reprogramming Neutral Lipid Metabolism in Mouse Dendritic Leucocytes Hosting Live *Leishmania amazonensis* Amastigotes

**DOI:** 10.1371/journal.pntd.0002276

**Published:** 2013-06-13

**Authors:** Hervé Lecoeur, Emilie Giraud, Marie-Christine Prévost, Geneviève Milon, Thierry Lang

**Affiliations:** 1 Institut Pasteur, Département de Parasitologie et Mycologie, Laboratoire Immunophysiologie et Parasitisme, Paris, France; 2 Institut Pasteur, Département Biologie Cellulaire et Infection, Plateforme de Microscopie Ultrastructurale, Paris, France; 3 Institut Pasteur, Département Infection et Epidémiologie, Laboratoire des Processus Infectieux à Trypanosomatidés, Paris, France; University of Notre Dame, United States of America

## Abstract

**Background:**

After loading with live *Leishmania (L) amazonensis* amastigotes, mouse myeloid dendritic leucocytes/DLs are known to undergo reprogramming of their immune functions. In the study reported here, we investigated whether the presence of live *L. amazonensis* amastigotes in mouse bone marrow-derived DLs is able to trigger re-programming of DL lipid, and particularly neutral lipid metabolism.

**Methodology/Principal Findings:**

Affymetrix-based transcriptional profiles were determined in C57BL/6 and DBA/2 mouse bone marrow-derived DLs that had been sorted from cultures exposed or not to live *L. amazonensis* amastigotes. This showed that live amastigote-hosting DLs exhibited a coordinated increase in: (i) long-chain fatty acids (LCFA) and cholesterol uptake/transport, (ii) LCFA and cholesterol (re)-esterification to triacyl-*sn*-glycerol (TAG) and cholesteryl esters (CE), respectively. As these neutral lipids are known to make up the lipid body (LB) core, oleic acid was added to DL cultures and LB accumulation was compared in live amastigote-hosting versus amastigote-free DLs by epi-fluorescence and transmission electron microscopy. This showed that LBs were both significantly larger and more numerous in live amastigote-hosting mouse dendritic leucocytes. Moreover, many of the larger LB showed intimate contact with the membrane of the parasitophorous vacuoles hosting the live *L. amazonensis* amastigotes.

**Conclusions/Significance:**

As leucocyte LBs are known to be more than simple neutral lipid repositories, we set about addressing two related questions. Could LBs provide lipids to live amastigotes hosted within the DL parasitophorous vacuole and also deliver? Could LBs impact either directly or indirectly on the persistence of *L. amazonensis* amastigotes in rodent skin?

## Author Summary

Once they have gained entry to mammals, live *Leishmania (L) amazonensis* amastigotes are known to subvert both macrophages and dendritic leucocytes (DLs) as host cells. These *L. amazonensis* amastigotes then may or may not proliferate in these two phagocytic leucocyte lineages, but in both cases the otherwise versatile differentiation program of these lineages is known to be rapidly remodeled. Here, we describe the rapid reprogramming of C57BL/6 and DBA/2 mouse bone marrow-derived DLs, with a special focus on cytosolic lipid bodies (LBs) that are known to store neutral lipids such as triacyl-*sn*-glycerol (TAG) and cholesteryl esters (CE). After extracting RNA from carefully sorted amastigote-free DLs and *L. amazonensis* amastigote-hosting DLs, an Affymetrix-based analysis clearly showed a singular and coordinated increase in DL transcripts involved in (i) long-chain fatty acid uptake, transport and esterification to TAG and (ii) cholesterol uptake and esterification to cholesteryl esters. Oleic acid was added to check that neutral lipid metabolism was both rapidly increased and reprogrammed in amastigote-hosting DLs. It should be noted that the LBs in live amastigote-hosting DLs were more numerous, and that the largest of these LBs were in contact with live amastigote- hosting parasitophorous vacuoles. We further discuss these findings in the context of live *L. amazonensis* amastigote-rodent host interactions.

## Introduction

*Leishmania* spp are protozoan parasites that are transmitted in the dermis of the mammalian host by blood-feeding sand flies. Once in the dermis of the mouse, the metacyclic promastigotes enter, both macrophages and dendritic leucocytes (DLs) where they differentiate into amastigotes within the parasitophorous vacuoles (PV). Though present only in low numbers in both skin and skin-draining lymph nodes, DLs have been recognized to play a central role in initiating and regulating the immune processes that take place in these two coupled tissues [1], [2], [3], [4]. More recently, the DL functional repertoire has been extended to cover metabolic functions such as the targeting of neutral lipids to lipid bodies (LBs) [5]. Briefly, not only have these cytosolic LBs been recognized in cell lineages other than the adipocyte lineage [6], [7], they are also known to perform functions beyond passive lipid storage and lipid homeostasis [8], [9], [10].

For instance, these LBs, which are composed of neutral lipids, i.e. triacyl-*sn*-glycerol (TAG) and cholesteryl esters (CE), have been observed in both non-myeloid cell lineages and myeloid leucocyte lineages. Human fibroblasts hosting *Toxoplasma gondii* [11], [12], or human erythrocytes hosting *Plasmodium falciparum* [13], historically and currently are under investigation to decipher LB biogenesis and functions [14]. These investigations have shown that tissue macrophages hosting parasitic microbes such as *Mycobacterium tuberculosis* [15], [16] and *Trypanosoma cruzi* [17] contain higher numbers of LBs. They also showed that the LBs in mammalian myeloid leucocytes have functions that range from the synthesis of inflammatory mediators [18] to the cross-presentation of phagocytosed antigens [19] and the delivery of lipids to the live parasitic intracellular microorganisms hosted by these myeloid leucocytes [16]. Altogether, these findings suggest that the biogenesis of these cytosolic LBs reflects recourse, by these parasitic intracellular microorganisms, to harbor host cell-derived lipids for their full development.

As mentioned above, although macrophages are the dominant myeloid leucocytes hosting cell-cycling *L. amazonensis (L. am)* amastigotes, the DLs hosting live *L. am* amastigotes are known to contribute to sustained remodeling of mouse dermis in at least some inbred strains as niches where small amastigote populations persist. Interestingly, while *L. am*-hosting C57BL/6, C3H/He and BALB/c skin (ear pinna or footpad) shows uncontrolled *L. am* amastigote burden and progressive skin damage [20], [21], [22], [23], [24], [25], [26], *L. am*-hosting DBA/2 mouse ear pinna shows all the features of natural rodent ear pinna, namely rapid control of the amastigote population [20] in a process that not only prevents the development of skin damage but could also account for the persistence of transmissible amastigotes to the blood-feeding adult female sand flies.

Given the low frequency of live amastigote-hosting DLs in both skin and skin-draining lymph nodes, we decided to begin our comparative analysis with C57BL/6 and DBA/2 bone marrow-derived DLs generated from GM-CSF-responsive progenitors. Once generated, the DL cultures were maintained at 34°C (mouse skin temperature) and further exposed or not to *L. am* amastigotes that had been carefully collected from nude mouse footpads inoculated with *L. am* promastigotes. Twenty-four hours later, both amastigote-free DLs and DLs hosting live *L. am* amastigotes were sorted from the four distinct cultures by flow cytometry in Biosafety Level 2 (BSL2) containment. This procedure yielded live *L. am* amastigote-hosting DLs and control DLs, both at high purity. These four distinct sorted DL populations were then subjected to a comparative high content analysis of their transcriptional profiles in order to detect any coordinated transcriptional profiles that could account for LB biogenesis and dynamic features [27]. Of the many transcripts that were seen to be upregulated, we focused on those coding for molecules that ensure i) the transport/uptake of non esterified fatty acids (NEFA) and the uptake of cholesterol, ii) the esterification of uptaken fatty acids and cholesterol iii) and those transcripts that regulate LB biogenesis and dynamic features. Finally, in this paper, we briefly discuss how cytosolic LBs that autonomously emerge in DLs hosting live *L. am* amastigotes could both directly and indirectly contribute - in damage-free rodent skin - to the sustained presence of *L. am* intracellular amastigotes that are transmissible to blood-feeding female sand flies.

## Materials and Methods

### Mice and ethics-related statement

Six-week-old female DBA/2, C57BL/6 and Swiss *nu/nu* mice were purchased from Charles River (Saint Germain-sur-l'Arbresle, France) and housed in our A3 animal facility in compliance with the relevant guidelines in force at Institut Pasteur which is a member of the “*Comité d'Ethique pour l'Expérimentation Animale*” (CEEA) - Ile de France. Animal housing conditions and the procedures used in the work described herein were approved by the “*Direction des Transports et de la Protection du Public, Sous-Direction de la Protection Sanitaire et de l'Environnement, Police Sanitaire des Animaux*” under number B75-15-28 in accordance with the Ethics Charter of animal experimentation that includes appropriate procedures to minimize pain and animal suffering. HL, TL and GM are authorized to perform experiments on vertebrates (licences: HL, 75-1550; GM, 75-331; TL, 75-717 issued by the Paris Department of Veterinary Services, DDSV) and were responsible for all the experiments conducted personally or under their supervision as governed by the laws and regulations relating to the protection of animals.

### Sampling and preparation of *L. am* amastigotes

*Ds*Red2 *transgenic L. am* were generated as previously described [27]. Wild-type or *Ds*Red2-transgenic *L. am* strain LV79 (WHO reference number MPRO/BR/72/M1841) amastigotes were prepared as previously reported [27]. Briefly, footpad skin hosting proliferative amastigotes was sampled in Swiss nude mice and amastigote populations further extracted and prepared for addition to DL cultures.

### Generation of mouse bone marrow-derived DLs and preparation of DL cultures exposed or not to live *L. am* amastigotes

DLs were differentiated from bone marrow cells of 6-week-old DBA/2 or C57BL/6 mice according to a method previously described [28]. Briefly, bone marrow cells were seeded at 4×10^6^ cells per 100 mm diameter bacteriological-grade Petri dish (Falcon, Becton Dickinson Labware, Franklin Lakes, NJ) in 10 ml of Iscove's modified Dulbecco's medium (IMDM; BioWhittaker Europe, Verviers, Belgium) supplemented with 10% heat-inactivated foetal calf serum (FCS; Dutscher, Brumath, France), 1.5% supernatant from the GM-CSF-producing J558 cell line, 50 U/ml penicillin, 50 µg/ml streptomycin, 50 µM 2-mercaptoethanol and 2 mM glutamine. Cultures were incubated at 37°C in a humidified gas phase with 18% O_2_ and 5% CO_2_. On day 6, suspended cells and loosely adherent cells were harvested using 1% Versene (EDTA) (Seromed) pre-warmed at 37°C and cultured in complete IMDM supplemented with 10% primary culture supernatant. On day 10, cells were harvested using 1% Versene (EDTA) pre-warmed at 37°C and distributed in hydrophobic 6-well plates (Greiner, St Marcel, France) at a concentration of 9×10^5^ cells/ml in 3 ml complete IMDM. On day 14, DL-containing plates were switched to 34°C and live *L. am* amastigotes were added - at a ratio of 5 amastigotes for 1 DL - or not for a further 24 hours in complete medium [29], or in complete medium supplemented with 200 µM oleic acid loaded onto bovine serum albumin (BSA) [30].

For further analyses by flow cytometric and epifluorescence microscopy (EFM), DLs were carefully detached after 5 minutes of incubation in 1% Versene (EDTA) solution at 34°C and resuspended at 4°C in Dulbecco's PBS with 2% FCS (PBS-FCS).

### Sorting, in BSL2 containment, of both (i) DL populations hosting live *L. am* amastigotes from DL cultures exposed to amastigotes and (ii) control DLs from cultures left unexposed to amastigotes

All experimental procedures were performed according to BSL2 practices. Cells were first incubated in PBS-FCS supplemented with 10% heat-inactivated donkey serum for 5 minutes. The cells were then incubated for 30 minutes in PBS-FCS containing 0.2 g/ml anti-MHCII monoclonal antibodies (mAbs) (M5/114) conjugated to PE-Cy5 (eBioscience, San Diego, USA). After two washes, cells were re-suspended at 5×10^6^ cells/ml in PBS containing 3% FCS and 1% supernatant from the GM-CSF producing J558 cell line [31]. Cell aggregates were dissociated on a 70 µm filter (Falcon), and placed on ice pending cell sorting as previously described [27]. Cell sorting was performed on a FACSAria (BD Biosciences, San Jose, CA) fitted with fully sealed sample injection and sort collection chambers operating under negative pressure. After staining with M5/114, DLs were selected by BD FACSDiva software (BD Biosciences). PE-Cy5 and *Ds*Red2 fluorescence was collected through 695/40 and 576/26 band pass filters, respectively. FSC and SSC were displayed on a linear scale and used to discard cell debris [27]. Live amastigote-hosting DLs were sorted by selecting cells expressing both surface MHC II molecules and *Ds*Red2 fluorescence. Sorting conditions included a sheath pressure of 70 Psi, a flow rate of 7 and the use of a 70 µm nozzle tip. Cells were collected at 4°C in polypropylene tubes (BD Biosciences) previously coated with FCS (overnight at 4°C). Sorted cells were immediately used for further studies [27].

### DL extracted RNA integrity control

Total RNA was extracted from MHC II^+^ DLs (RNeasy+ Mini-Kit, Qiagen) and its quality and concentration was determined using a NanoDrop ND-1000 micro-spectrophotometer (Kisker, http://www.kisker-biotech.com) and an Agilent-2100 Bioanalyzer (Agilent, http://www.chem.agilent.com). RNA Integrity Number (RIN) scores were determined for each sample (RNA Integrity Numbers ≥7.5) providing an objective and standardised measure of RNA quality on a scale of 1 to 10 (with a value of 10 corresponding to the highest quality) [32], [33].

### GeneChip hybridization and data analysis

Altogether, 200 ng of total RNA per sample were processed, labelled and hybridized to Affymetrix Mouse Gene ST 1.0 arrays, following Affymetrix Protocol (http://www.affymetrix.com/support/downloads/manuals/expression_analysis_technical_manual.pdf). Three Biological replicates per condition were run as described previously [20]. After hybridization, the arrays were stained and scanned at 532 nm using an Affymetrix GeneChip Scanner 3000 which generated individual CEL files for each array. Gene-level expression values were derived from the CEL file probe-level hybridization intensities using the model-based Robust Multichip Average algorithm (RMA) [34]. RMA performs normalization, background correction and data summarization. An analysis was performed using the LPE test [35] (to identify significant differences in gene expression between parasite-free and parasite-harbouring DLs), and a p-value of p<0.05 was considered as significant. Estimated false discovery rate (FDR) was calculated using the Benjamini and Hochberg approach [36] in order to correct for multiple comparisons. A total of 1,340 probe-sets showing significant differential expression were input into Ingenuity Pathway Analysis software v5.5.1 (http://www.ingenuity.com), to perform a biological interaction network analysis. The symbols of the modulated genes are specified in the text (fold change [FC] values between brackets), while their full names are given in additional file 1. MIAME-compliant data are available in the GEO database http://www.ncbi.nlm.nih.gov/geo/ accession GSE.

### Multiparametric analyses by flow cytometry

Flow cytometric (FCM) analyses were performed on freshly detached cells. LBs were stained by adding 4,4-difluoro-1,3,5,7-tetramethyl-4-bora-3a,4a-diaza-*s*-indacene-8-propionic acid, succinimidyl ester, or BODIPY 493/503 (Molecular Probes, Life technologie SAS, Saint Aubin, France) to a concentration of 2 µg/ml in PBS for 30 minutes at 34°C. After careful washings in PBS, the presence of MHC II molecules was determined by staining.

The uptake of fluorescent palmitic acid (4,4-difluoro-5,7-dimethyl-4-bora-3a, 4a-diaza-s-indacene-3-hexadecanoic acid, BODIPY FL C16, Molecular Probes) was analysed by incubating DLs with 0.05 µM probe for 30 minutes at 34°C. Cells were washed as described above and the presence of MHC II molecules was detected by immune-staining. Briefly, DLs were incubated with the immuno-staining agent for 15 minutes in PBS-FCS supplemented with 10% heat-inactivated donkey serum. The DLs were then incubated in PBS containing 10% FCS and 0.01% sodium azide (NaN_3_) in the presence of antibodies directed against surface antigens. Extracellular staining procedures were performed with mAbs directed against MHC II (M5/114 clone) and CD36 (clone 72.1) conjugated to PE-CY5 and PE, respectively. Biotinylated antibodies directed against CD11c (HL3) and IgG control (B81-3 clone) were purchased from eBioscience and were used at 0.5 µg/ml. Streptravidin conjugated to Phycoeythrin/PE (Molecular Probes) was added (1.5 µg/ml) to detect binding of the biotinylated antibodies to their respective molecular targets. Intracellular amastigotes in PE MHC II-stained DLs were co-detected by intracellular immunophenotyping using 2A3-26 mAb [27] after adding Cytofix/Cytoperm solution (BD Biosciences). Intracellular staining of amastigotes in PE MHC II-stained DLs was performed after fixation in PBS containing 1% paraformaldehyde (PFA) for 20 min at 4°C. DLs were then washed in Perm/Wash solution from the BD Cytofix/Cytoperm Plus Kit (BD Biosciences) and incubated for 30 min at 4°C with 5 µg/ml of Alexafluor 488- conjugated 2A3-26 mAb which was shown to strictly bind to the *L. amazonensis* amastigote [29].

### Multiparametric analyses by EFM of DL surface molecules and cytosolic LBs

BODIPY FL C16 and BODIPY 493/503 were used both to measure fatty acid (FA) uptake by DL and detect the presence of LBs in DL cytosol.

After detachment, DLs were centrifuged on poly-L-lysine-coated glass coverslips at 1000 rpm for 5 minutes then incubated at 34°C for 1 hour. FA uptake and localization was analysed after a short 30-minute incubation with BODIPY FL C16 (0.05 µM in PBS) at 34°C. Samples were then washed and fixed in 2% PFA, surface MHC II molecules were stained as described above. LBs were stained in PBS containing 2 µg/ml BODIPY 493/503 for 30 minutes at 34°C. Samples were then washed in PBS and fixed in 2% PFA for 20 minutes. The presence of surface MHC II molecules was then detected as described above using biotinylated M5/114 antibodies (e-Bioscience) revealed by streptavidin Texas red (Pierce, Thermo Scientific, Rockford, USA).

Finally, samples were mounted on glass slides with Mowiol (Calbiochem, Darmstadt, Germany) containing 5 µg/ml Hoechst 33342 (Molecular Probes): as the latter is incorporated into DNA it stains the nuclei of both the host cells and the amastigotes. Images were acquired on an upright Zeiss Axioplan 2 microscope controlled by Zeiss Axiovision 4.4 software.

### DL culture handling and processing for transmission electron microscopy (TEM)

DLs intended for conventional TEM analyses were cultured for 24 hours post contact with amastigotes or not, then carefully detached. After washing in PBS, DLs were fixed overnight at 4°C in 2.5% glutaraldehyde buffered with 0.1M sodium cacodylate buffer, pH 7.2. DLs were then washed three times in the same buffer, and were post-fixed in 2% osmium tetroxide (OsO4) (Merck, Germany) in 0.1M sodium cacodylate buffer, pH 7.2, for 1 hour. After dehydration in graded series ethanol, they were embedded in Epon 812 mixture and subjected to heat polymerization. Thin sections were then cut on a Leica-Microsystems UC7 ultramicrotome, collected on 200 mesh formvar-coated cupper grids and stained with uranyl acetate and Reynold's lead citrate, then observed on a JEM 1200 EXII electron microscope (Jeol, Tokyo, Japan) using a megaview camera (Olympus, Soft imaging systems, Münster, Germany).

Samples for high pressure freezing and freeze substitution were inactivated by aldehyde fixation as described above. Cells were then taken up in capillary tubes (Leica, Vienna) as described [37]. The filled tube was separated by clamping into segments shorter than 2 mm and was then placed in the 200 µm deep cavity of a brass planchette, Type A (Agar Scientific, Stanstad, UK) filled with 1-hexadecen. The flat side of the complementary Type B planchette closed the filled planchette and it was frozen in an HPM 010 unit (BalTec, now Abra Fluid AG, Widnau, Switzerland). Freeze substitution was performed in an osmium (2% OsO4) acetone medium with 1% of water being added [38]. Gently pressing a pre-cooled forceps (No. 5, Dumont, Switzerland) in liquid nitrogen introduced small fractures into the hexadecen and thus allowed the substitution mix access to the sample. Substitution was carried out at −90°C for 48 hours and at −60°C and −30°C for 8 hours each in a freeze substitution device (Leica, Vienna, Austria). Samples were then incubated for 1 hour on ice in 2% OsO4 in dry acetone, followed by 1 h at room temperature in the same solution. Samples were thereafter washed with dry acetone and embedded stepwise in EPON.

### Statistical analyses

Two-sided Student's paired t-tests were used to compare flow cytometry experiments (4<n<6). A Mann-Whitney test was used to compare ear thickness measurements and number of parasites per DL.

## Results and Discussion

### C57BL/6 and DBA/2 DLs hosting live *L. am* amastigotes show only minor transcriptional modifications compared to control DLs

DLs were derived from GM-CSF-responsive progenitors (otherwise known to be present in the bone marrow cell suspensions prepared from the femurs of C57BL/6 and DBA/2 mouse inbred strains) as previously described for DLs derived from BALB/c mouse bone marrow [27]. More than 97% of the cells in these cultures expressed CD11c in parallel to CD11a and CD11b (data not shown). Two different cell subsets were defined by flow cytometry (FCM) analysis. The first did not express surface MHC II molecules, and consequently was not considered as *bona fide* DLs (subset 1, figure S1A). The second did express surface MHC II molecules, albeit at various intensities. These are DL phenotypic signatures per se (subset 2, figure S1A). The latter features were thus used for all subsequent DL FCM analyses, as well as for DL sorting.

The cultures were either left in medium alone (ctrl condition) or places in contact with live *L. am* amastigotes at a ratio of 5 amastigotes per cell (*L. am* condition) and either processed or analysed 24 hours later. Combined with the DL features mentioned above, transgenic *L. am* amastigotes expressing fluorescent *Ds*Red2 protein were used for the specific detection and sorting of all DLs harbouring live *L. am* amastigotes, without further fixation and permeabilization. Thus, ctrl DLs and DLs hosting live *Ds*Red2 *L. am* amastigotes were sorted based on their expression of MHC II molecules and MHC II molecules plus *Ds*Red2 fluorescence, respectively (Figure S1B). This sorting strategy constitutes a major improvement over all previously published methods for an in-depth comparative characterization of ctrl DLs versus DLs harbouring live *Ds*Red2 *Leishmania* amastigotes (Figure S1B) [27].

A genome-wide transcriptional analysis based on Affymetrix technology was performed on sorted DLs from both mouse strains. It highlighted that DLs hosting live *L. am* amastigotes showed only minor transcriptional modifications compared to ctrl DLs, and this both in term of frequency and magnitude. In fact, out of 28, 853 mouse genes, only 858 and 932 were captured with differential expression at the 5% significance level in C57BL/6 and DBA/2 DLs, respectively. These numbers correspond to only 1.6% and 1.8% of genes modulated in C57BL/6 and DBA/2 DLs harbouring live *L. am* amastigotes.

### Transcriptional signatures accounting for FA metabolism in mouse C57BL/6 and DBA/2 DLs hosting live *L. am* amastigotes

In the absence of any signatures indicative of *de novo* FA synthesis, the modulation of two transcripts, namely i) *phosphatidic acid phosphatase type 2B (ppap2B)* - known to code for a non-specific phosphatase located in plasma membrane lipid rafts and caveolae - and ii) *mgl* - known to convert monoacyl *sn* glycerol (MAG) into glycerol and NEFAs - was interpreted as signatures accounting for NEFA delivery in DLs hosting *L. am*+ also written *L. am^+^*DLs (Table 1 and figure S2). Therefore, any subsequent protein translation from the coordinated up-regulation of both i) scavenger receptor-coding transcripts and ii) NEFA-esterifying enzyme-coding transcripts, NEFA could be directed from the plasma membrane either to the cytosol or to the cortical Endoplasmic Reticulum (ER) where it could be enzymatically converted into chemically inert TAGs and cholesterol esters. The latter are known to be the core lipids stored in more or less numerous LBs, especially if lipolysis machinery is prevented from operating in these otherwise dynamic cytosolic LBs.

**Table 1:** Transcriptional signatures accounting for fatty acid metabolism in dendritic leucocytes hosting live *L. amazonensis* amastigotes.

Abbreviation	Name	Transcript cluster ID	FC Affymetrix		*p* value	
			C57BL/6	DBA/2	C57BL/6	DBA/2
**De novo fatty acid synthesis and metabolism**						
***acsl5***	acyl-CoA synthetase long-chain family member 5	10464045	**/**	**−1.62**	**NS**	**2.40E-2**
***fads3***	fatty acid desaturase 3	10134600	**/**	**−1.63**	**NS**	**6.03E-4**
***hlcs***	holocarboxylase synthetase	10441038	**−1.63**	**/**	**1.04 E-2**	**NS**
***nr1h3***	Nuclear receptor subfamily 1, group H, member 3	10484987	**+1.67**	**/**	**2.2 E-2**	**NS**
***scd1***	stearoyl-coenzyme A desaturase 1	10467979	**+1.65**	**/**	**4.47 E-3**	**NS**
**Fatty acid uptake and transport**						
***cav1***	caveolin-1	10536499	**+1.69**	**/**	**4.71E-02**	**NS**
***cav2***	caveolin-2	10536494	**+1.96**	**+2.13**	**3.41E-02**	**4.38E-03**
***cd36***	CD36 antigen	10528207	**+2.67**	**+3.96**	**5.95E-07**	**<1E-13**
***fabp4***	fatty acid binding protein 4	10497265	**+4.33**	**+2.68**	**>1E-13**	**6.48E-07**
***fabp5***	fatty acid binding protein 5	10490838	**+2.27**	**+4.66**	**7.25E-07**	**>1E-13**
***pparγ***	Peroxysome proliferator-activated receptor γ	10540897	**+1.67**	**+3.02**	**3.01E-03**	**3.09E-08**
***slc27a1***	solute carrier family 27, member a1	10572647	**/**	**+1.69**	**NS**	**4.29 E-2**
**Other processes**						
***dgat2l3***	diacylglycerol O-acyltransferase 2-like 3	10600988	**+2.22**	**+2.81**	**1.26E-02**	**4.88E-06**

The fold changes (FC) indicated were obtained by Affymetrix analysis and correspond to the difference between i) sorted live *L. amazonensis amastigote*-hosting DLs and ii) sorted control (unexposed to amastigotes) DLs. Significance (*p* value) is indicated for each transcript and each mouse strain.

### Long Chain Fatty Acid (LCFA) uptake/transport by/in live *L. am^+^* DLs

**Figure 1:**
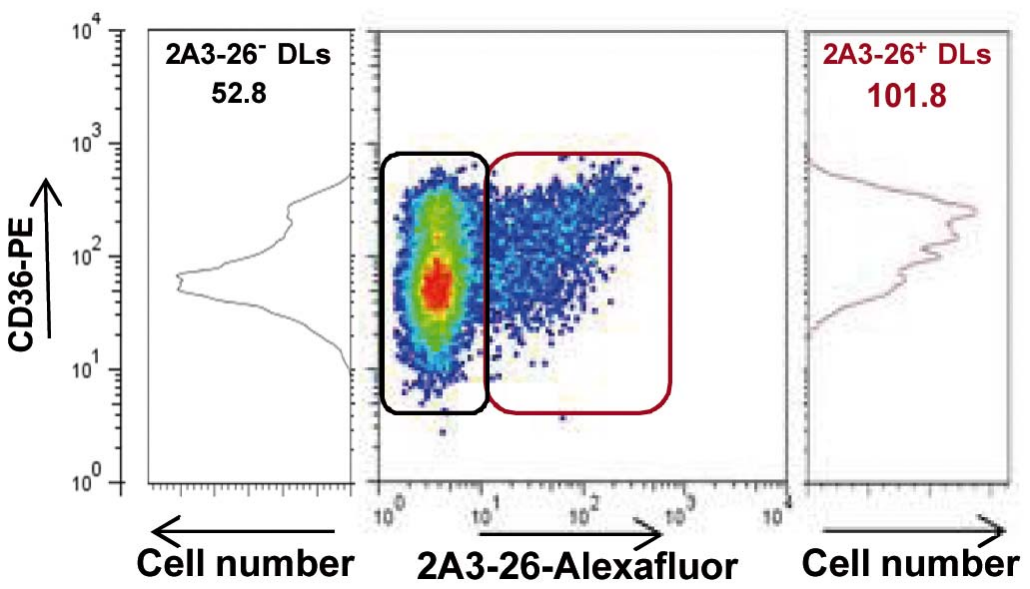
Flow cytometric analysis of CD36 expression in C57BL/6 DLs hosting live *L. am* amastigotes. *L. am* amastigotes were added to DL cultures at a ratio of 5 amastigotes per DL. Twenty-four hours later, DLs were detached and stained successively with anti- MHC II- PE-Cy5 and CD36-PE conjugated mAbs. As the fluorescence of transgenic *Ds*Red2 amastigotes was quashed by the fixation step, we used 2A3-26 mAb generated in our laboratory to image intact amastigotes inside their parasitophorous vacuoles. Thus, after fixation, the DLs were first permeabilized then labelled with alexafluor480-conjugated 2A3-26 mAbs before being analysed by FCM. This analysis was performed on gated MHC II^+^ DLs. The central dot plot shows CD36 expression and the presence of intracellular parasites. The profiles of CD36 expression and mean CD36 fluorescence value are shown for 2A3-26^+^ DLs (red gate) and 2A3-26^−^ DLs (black gate).

LCFA uptake was promoted in *L. am^+^*DLs by the concerted up-regulation of transcripts encoding for key surface molecules (Table 1). First, LCFA uptake may be promoted by an increase in transcripts encoding for fatty acid translocase (*cd36*), resulting in higher levels of CD36 in DLs harboring *L. am* amastigotes. This higher CD36 expression was confirmed at the protein level by FCM analyses (n = 4 independent experiments). The cell surface of *L. am* ^+^ DLs in mice of both inbred strains showed a higher quantities of CD36 than *L. am* ^−^ DLs (MFI CD36 for *L. am^−^* vs *L. am^+^* DLs: 36.5+/−23.0 versus 65.2+/−51.7 for C57BL/6 DLs, and 125.9+/−103.4 versus 157.4+/−78.6 for DBA/2 DLs, see figure 1 illustrating one of the 3 experiments). Second, the concomitant up-modulation of transcripts encoding for CD36 and caveolin-1 – one of the structural proteins of caveolae where CD36 can be targeted - may account for the increased LCFA uptake by live amastigote-hosting DLs. Interestingly in DBA/2 DLs, LCFA uptake and transport could also have been promoted by the up-regulation of the fatty acid transporter FATP1 (slc27a1) that probably facilitates LCFA uptake coupled with an esterification step (Table 1). *L. am^+^* DLs also showed up-modulation of transcripts encoding for the intracellular lipid chaperones FABP4 and FABP5 (Figure S2 and table 1) which are known to be involved in the uptake and metabolism of intracellular LCFA. The up-modulation of these transcripts probably resulted from up-modulation of the nuclear receptor PPAR-gamma (pparγ) (Table 1): PPAR-γ transcription factor is known to promote the expression of CD36, caveolin 1, FATP1 and FABP4, and each of these may further increase LCFA transport/uptake.

### Long Chain Fatty Acid (LCFA) delivery to both DL and live *L. am* amastigotes hosted within DL parasitophorous vacuoles (PV)

To determine whether DLs hosting live amastigotes show increased LCFA uptake, ctrl and *L. am* ^+^ cultures were loaded for 30 minutes with fluorescent palmitic acid (Bodipy FL-C16), and analysed by FCM (Figure S3A) and EFM (Figure S3B). Live *L. am* ^+^ (*Ds*Red2^+^) DLs showed higher fluorescence of BODIPY FL-C16 than *L. am^−^* (*Ds*Red2^−^) DLs. Interestingly, EFM analyses also revealed that LCFA were able to reach the amastigotes (Figure S3B insert), indicating that live amastigotes in their PV are capable of rapidly scavenging LCFA from their host cell.

### DLs hosting live *L. am* amastigotes contain enzymes that promote TAG generation

**Figure 2:**
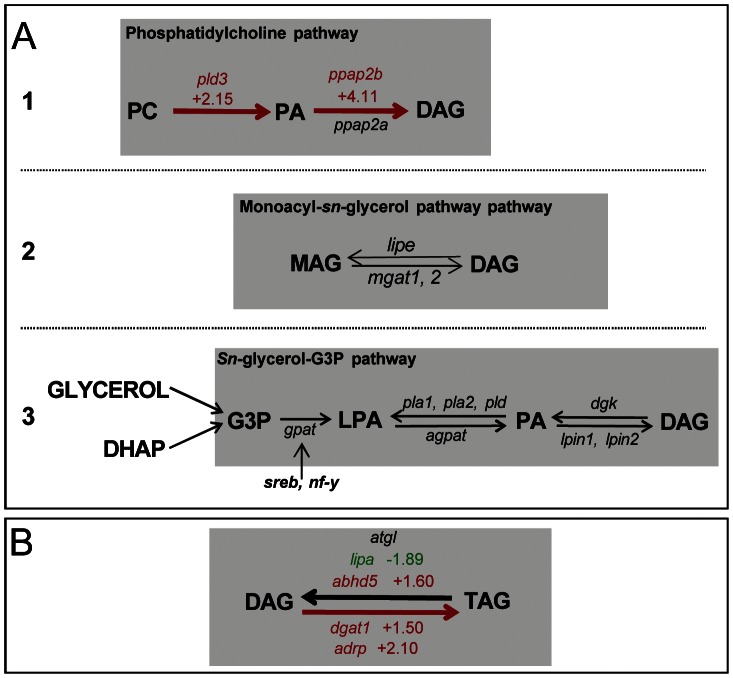
C57BL/6 DLs hosting live *Ds*Red2 *L. am* amastigotes showing transcriptional signatures for both DAG and TAG accumulation. Affymetrix-based analysis detected transcriptional signatures for both DAG and TAG accumulation. Up and down modulations observed between *L. am* hosting and control DLs are indicated in red and green respectively, for the three pathways leading to DAG/TAG accumulation. **Panel A: Different pathways leading to DAG accumulation.** **A1. Transcriptional signatures accounting for the phosphatidylcholine pathway** PC: Phosphatidylcholine, *ppar*γ: peroxysome proliferator-activated receptorγ, *pld3*: phospholipase D3, *ppab2b*: phosphatidic acid phosphatase type 2B. **A2. Monoacylglycerol pathway** MAG: monoacylglycerol, DAG: diacylglycerol, *mgat*1 and 2: monoacylglycerol O-acetyl transferases 1 and 2. **A3.** ***Sn*** **-glycerol-3P pathway** G3P: glycerol-3-phosphate, DHA: dihydroxyacetone-phosphate, LPA: Lysophosphatidic Acid, PA: Phosphatidic Acid, *sreb*: sterol regulatory element binding protein, *nf-y: nuclear factor-Y*; *gpat*: glycerol-3-phosphate acyltransferase, *pla1*: phospholipase A1, *pla2*: phospholipase A2, *pld*: phospholipase D, *agpat*: 1-acyl-glycerol-3-phosphate acyltransferase, *dgk*: diacylglycerol kinase, *lpin 1*: lipin-1, *lpin2*: lipin-2. **Panel B: DAG to TAG conversion.** *abhd5*: α/β hydrolase domain-containing protein 5, *atgl*: adipose triglyceride lipase, *dgat1*: acyl CoA:diacylglycerol acyltransferase 1, *dgat2*: acyl CoA:diacylglycerol acyltransferase 2, *dgat2L3*: diacylglycerol O-acyltransferase 2-like 3, *adrp*: adipose differentiation related protein, *lipa*: lysosomal acid lipase A.

Glycerolipids are the main structural and functional constituents of *Leishmania* membranes, with TAG being the second most prevalent lipid class in axenically cultured *Leishmania* promastigotes [39]. Our Affymetrix-based analyses showed that these enzymes were subject to transcriptional modulations that may promote several pathways involved in diacyl-*sn*-glycerol (DAG) homeostasis and TAG generation (Table 2, figure 2).

**Table 2:** Transcriptional signatures accounting for TAG metabolism in dendritic leucocytes (DLs) hosting live amastigotes.

Abbreviation	Name	Transcript cluster ID	FC Affymetrix		*p* value	
			C57BL/6	DBA/2	C57BL/6	DBA/2
***abhd5***	α/β hydrolase domain-containing protein 5	10590452	**+1.60**	**+1.67**	**3.94E-03**	**1.34E-2**
***acat2***	acetyl-CoA acetyltransferase 2	10447891	**+1.58**	**/**	**2.21E-2**	**NS**
***adrp***	adipose differentiation related protein	10514221	**+2.10**	**+2.34**	**3.11E-08**	**5.24E-07**
***agpat7***	1-acylglycerol-3-phosphate O-acyltransferase 7	10474526	**/**	**−1.54**	**NS**	**3.03E-2**
***aqp3***	aquaporin 3	10512156	**−1.81**	**/**	**5.5E-04**	**NS**
***aqp9***	aquaporin 9	10594825	**−1.65**	**/**	**1.3E-02**	**NS**
***cebpβ***	CCAAT/enhancer binding protein beta	10478890	**+2.23**	**+3.00**	**5.26E-6**	**3.13E-12**
***dgat1***	diacylglycerol O-acyltransferase 1	10429926	**+1.50**	**/**	**2.76E-02**	**NS**
***dgat2l3***	diacylglycerol O-acyltransferase 2-like 3	10600988	**+2.22**	**+2.81**	**1.26E-02**	**4.88E-06**
***lipa***	lysosomal acid lipase A	10467139	**−1.89**	**/**	**1.21E-04**	**NS**
***lipe***	lysosomal acid lipase E	10561031	**/**	**−1.60**	**NS**	**1.7169E-2**
***lipf***	lipase, gastric lipf	10462542	**+3.3**	**/**	**4.80E-09**	**NS**
***lpl***	lipoprotein lipase	1057213	**/**	**+2.28**	**NS**	**2.64e-06**
***mgll***	monoglyceride lipase	10539894	**+1.90**	**+2.27**	**1.92E-05**	**2.91E-06**
***pld3***	phospholipase D family, member 3	10561306	**+2.15**	**+2.93**	**8.43E-07**	**4.72E-10**
***pld4***	phospholipase D family, member 4	10398907	**/**	**+1.98**	**NS**	**2.26E-03**
***ppap2b***	phosphatidic acid phosphatase type 2B	10506496	**+4.11**	**+4.10**	**3.74E-10**	**1.39E-12**
***ppap2c***	phosphatidic acid phosphatase type 2C	10370552	**−1.53**	**/**	**2.76E-0.2**	**NS**
***pparγ***	Peroxysome proliferator-activated receptor γ	10540897	**+1.67**	**+3.02**	**3.01E-03**	**3.09E-08**
***smpdl3a***	sphingomyelin phosphodiesterase, acid-like 3A	10363231	**+2.19**	**+3.96**	**5.05E-04**	**8.65E-12**
***stom***	stomatin	10482030	**+1.73**	**/**	**9.11E-4**	**NS**

The fold changes (FC) indicated were obtained by Affymetrix analysis and correspond to the difference between sorted i) live *L. am.* amastigote-hosting DLs and ii) sorted control (unexposed to amastigotes) DLs. Significance (*p* value) is indicated for each transcript and each mouse strain.

### The enzymes promoting DAG production in live *L. am^+^* DLs hosting

We then analysed the pathways that lead to DAG production by considering two sites of potential production, namely, i) plasma membrane and ii) endoplasmic reticulum. DAG production may be promoted in the plasma membrane by up-modulation of non-specific phosphatidic acid phosphatase type 2B (*ppap2b*) and phospholipases d (*pld3*, *pld4*, figure 2A1, table 2). These enzymes may act sequentially in lipid rafts to generate DAG from phosphatidylcholine, as shown previously for *pld2* and *ppap2b* [40]. Whether or not the DAG produced here in the plama membrane level can rapidly participate in TAG generation will need further analysis of the presence or not of cortical ER, i.e., ER contacting the DL plasma membrane. Non-cortical ER is not expected to be a site of DAG production for first, although we cannot exclude the possibility that MAG conversion may be favoured by up-modulation of *dgat1* in C57BL/6 DLs (Table 2), MAG esterification into DAG (MAG pathway) is not promoted (Figure 2A2, table 2). Second, in contrast to the modulation of the transcripts accounting for DAG conversion to TAG - that will be further documented in the next section - no up-regulation was noted for either of the transcripts coding for the enzymes involved in the *de novo sn*-glycerol-3-phosphate pathway (Figure 2A3), or the transcripts coding for the enzymes involved in DAG conversion to other molecules such as MAG, PC, phosphatidic acid or phosphatidylethanolamine.

### Increased TAG generation may predominate over TAG hydrolysis, at least in C57BL/6 DLs hosting live *L. am* amastigotes

Not only may increased TAG generation in the caveolae [41] be sustained by increased NEFA uptake and re-esterification, but the up-regulation of *dgat1* transcripts (Figure 2B, table 1) may also account for the DAG conversion to TAG. We also noted up-regulation of the transcripts encoding for ADRP (perilipin-2), which is known to coat the surface of lipid droplets and protect TAG from the action of lipases. By contrast, we noted the up-modulation of *abhd5* transcripts (Figure 2B, table 2). These encode for a lysophosphatidic acid acyltransferase which is able to increase 20-fold the hydrolase activity of *atgl* [42]. Consequently, a certain amount of TAG could be hydrolysed in *L. am* ^+^ DLs to balance NEFA re-esterification. The increased *dgat1* transcripts observed in C57BL/6 DLs suggests that such TAG hydrolysis occurs since this enzyme would appear to be involved in the re-esterification of hydrolysed TAG by exogenous FA [43]. This complex process may lead to a remodelling of TAG by preferring substrates such oleoyl-CoA. In C57BL/6 DLs, the down-modulation of *lipa* transcripts is expected to prevent any hydrolysis of the TAG stored in LBs (see next section).

### Cholesterol uptake and generation of cholesteryl ester

**Figure 3:**
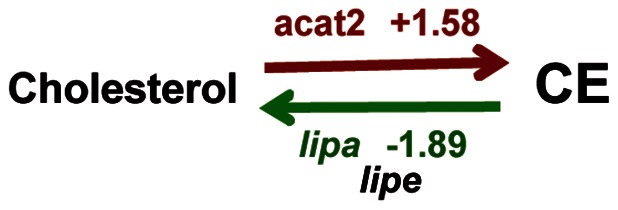
C57BL/6 DLs hosting live *Ds*Red2 *L. am* amastigotes showing cholesterol storage transcriptional signatures. Affymetrix-based analysis detected DL transcriptional signatures of cholesterol storage. *acat2*: acetyl-CoA acetyltransferase 2, *lipa*: lysosomal acid lipase A.

As *Leishmania* perpetuation is known to be strictly reliant upon cholesterol provided by the host organism, we set about investigating whether cellular cholesterol uptake, trafficking and the complex systems regulating cholesterol delivery may be modified in DLs hosting live *L. am* amastigotes. Mammalian cells acquire cholesterol from three sources: i) from *de novo* synthesis in the ER, iii) from low-density lipoprotein (LDL)-derived cholesterol and iii) from the cholesterol/cholesteryl ester (CE) cycle. Our data showed that, in contrast to BALB/c mouse bone marrow-derived macrophages hosting proliferating *L. am* amastigotes [44], DLs harbouring live *L. am* amastigotes did not show any fluctuations in the many enzyme-coding transcripts involved in the *de novo* biosynthesis of cholesterol. The only exception to this was *mvd* in DLs of C57BL/6 origin (Table 3). We noted that cholesterol hydroxylase, which is known to convert cholesterol to 25-hydroxycholesterol/25-HC, was up-regulated. It should here be noted that 25-HC can act on the SCAP/SREBP complex - involved in cholesterol synthesis - by suppressing the SREBP maturation process [45], [46], and that any increase in 25-HC might prevent cholesterol synthesis. By contrast, we also noted that transcripts encoding for molecules involved in the uptake and transport of exogenous cholesterol and cholesterol storage were up-modulated (Table 3). For instance, *L. am* ^+^ DLs showed concerted up-modulation of multiple surface receptors (Table 3) that may promote the uptake of cholesterol, either specifically or after LDL endocytosis. Cholesterol esterification in *L. am* ^+^ C57BL/6 DLs appeared to be promoted through increased transcription of cytosolic thiolase *acat2* (Figure 3, Table 3). This increased CE generation may also be promoted by the higher FA content resulting from increased FA uptake and transport, as described previously. The absence of any modulation of *aadacl* transcript encoding for cholesteryl ester hydrolase (CEH), and the down-modulation of *lipa* and *lipe* in *L. am* ^+^ C57BL/6 and DBA/2 DLs, respectively, (Table 3) suggested that CE hydrolysis is prevented. No transcriptional signatures were detected except the increased transcription of *cav-1* that could account for cholesterol delivery to cell surface caveolae and promote its efflux out of the *L. am* ^+^ DLs.

**Table 3:** Transcriptional signatures accounting for cholesterol metabolism in dendritic leukocytes (DLs) hosting live *L. amazonensis* amastigotes.

Abbreviation	Name	Transcript cluster ID	FC Affymetrix		p value	
			C57BL/6	DBA/2	C57BL/6	DBA/2
***De novo synthesis of cholesterol***						
***ch25h***	cholesterol 25-hydroxylase	10467136	**+2.84**	**+3.09**	**6.19E-10**	**5.66E-10**
***mvd***	mevalonate (diphospho) decarboxylase	10582310	**+1.72**	**/**	**3.15 E-3**	**NS**
***Uptake and transport of cholesterol***						
***cav1***	caveolin-1	10536499	**+1.69**	**/**	**4.71E-02**	**NS**
***cd68***	CD68 antigen	10387536	**+1.72**	**+2.56**	**4.50E-3**	**9.59E-9**
***cd91***	CD91 antigen	10373223	**+2.51**	**+3.20**	**5.92E-7**	**9.59E-9**
***cd204***	CD204 antigen,	10578264	**+2.17**	**+2.15**	**4.2E-4**	**2.69E-5**
***marco***	macrophage receptor with collagenous structure	10357261	**+4.4**	**+3.11**	**<1E-13**	**1.08E-8**
***olr1***	oxidized low density lipoprotein (lectin-like) receptor 1	10548385	**+2.37**	**+2.66**	**2.77E-5**	**1.26E-5**
***Storage of cholesterol***						
***acat2***	acetyl-CoA acetyltransferase 2	10447891	**+1.58**	**/**	**2.21E-2**	**NS**
***lipa***	lysosomal acid lipase A	10467139	**−1.89**	**/**	**1.21E-04**	**NS**
***lipe***	lysosomal acid lipase E	10561031	**/**	**−1.60**	**NS**	**1.7169E-2**

The fold changes (FC) indicated were obtained by Affymetrix analysis and correspond to the difference between i) sorted live *L. amazonensis* amastigote-hosting DLs and sorted control (unexposed to amastigotes) DLs. Significance (*p* value) is indicated for each transcript and each mouse strain.

### DLs hosting live *L. am* amastigotes contain more cytosolic LBs than control DLs

**Figure 4:**
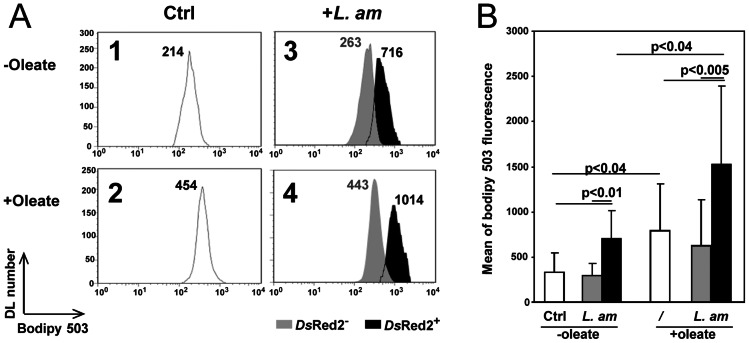
FCM detection of lipid bodies (LBs) in control C57BL/6 DLs and C57BL/6 DLs hosting *Ds*Red2 *L. am* amastigotes. **Panel A. Representative analysis of the fluorescence of BODIPY 493/503 in MHC II^+^ DLs.** A1, A2. Control C57BL/6 DL cultures –i.e. not exposed to *L. am* amastigotes- were incubated or not at 34°C with 200 µM oleate for 21 hours. A3, A4. *Ds*Red2-*L. am* were added to DL cultures at a ratio of 5 *Ds*Red2-*L. am* per DL (+*L. am*). Oleic acid (200 µM) was added 3 hours post inoculation, (A4: +Oleate) or not (A3: −Oleate) for a further 21 hours at 34°C. Control and *L. am*-loaded DLs were then detached. LBs were stained with BODIPY 493/503 2 µg/ml in PBS for 30 minutes at 34°C. The cells were then incubated with anti MHC II- PE-Cy5 mAb to analyze only the MHC class II-positive DLs. FCM histograms show BODIPY 493/503 fluorescence for Ctrl (A1, A2: white histograms) and *Ds*Red2 *L. am*-hosting DLs (A3, A4) (*Ds*Red2^+^, black histograms) and amastigotes-free DLs (*Ds*Red2^−^, grey histograms). **Mean fluorescence intensity was indicated for each histogram.** **Panel B: Statistical analysis of the BODIPY 493/503 MFI values.** Mean BODIPY 493/503 fluorescence by MHC II^+^ DLs was determined for n = 7 independent experiments. MFI in *L. am*-loaded cultures is shown for *Ds*Red2^+^ and *Ds*Red2^−^ DLs as black and grey bars, respectively. Statistical analyses were performed by the Mann Whitney test.

**Figure 5:**
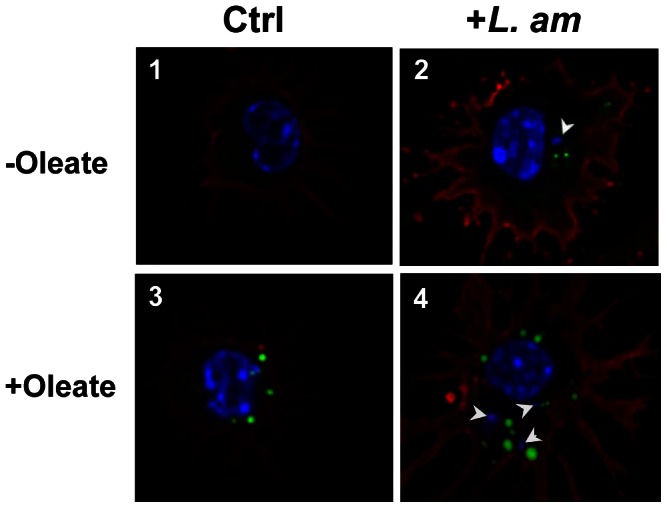
EFM detection of lipid bodies (LBs) in C57BL/6 DLs. Controls (left panels) and *L. am* amastigote-loaded DLs (right panels) were incubated or not with oleate as described previously. Cells were then detached, deposited on coverslips, and LBs were evidenced by the incorporation of BODIPY 493/503 2 µg/ml in PBS for 30 minutes at 34°C (in green). Cells were fixed and stained with M5/114 mAb (in red). DL and amastigote nuclei were stained with Hoechst 33,342 (blue spots indicated by arrowheads).

We noted increased transcriptional expression of genes coding for LB surface proteins, in particular *cav-1*, *cav-2*, *stom*, *abhd5* and *adrp*. Since no evidence was found of any promotion of *de novo* synthesis of cholesterol and TAG, we cultured DLs hosting or not hosting live *L. am* amastigotes in the presence of oleic acid – a mono-unsaturated LCFA - bound to BSA and compared LB number and size in *L. am* ^+^ versus *L. am^−^* DLs. Briefly, DLs were places in contact with *Ds*Red2-LV79 amastigotes for 3 hours and cultured in the presence of 200 µm oleate/BSA for the next 21 hours. Thirty minutes before FCM analyses of unfixed control (Ctrl) and *L. am^+^* DLs, a fluorescent lipophilic probe - BODIPY 493/503 dye - was added to the cultures: its incorporation into the highly hydrophobic ester core of LBs allowed the latter to be evidenced by FCM or EFM as green organelles (Figures 4 and 5). Parasitized DLs were detected by the orange fluorescence of intracellular *Ds*Red2^+^ amastigotes. Figure 4A shows the outcome of a representative FCM experiment performed on C57BL/6 mice, and Figure 4B the collective results of 7 independent experiments. Similar results were obtained for DBA/2 DLs (data not shown). White histogram (Figure 4A1) represents background mean fluorescence emitted by ctrl DLs (here mean fluorescence intensity (mfi) was 214). This value was increased (Figure 4A3, mfi = 716, black histogram) in the oleate-free DLs containing *Ds*Red2^+^ amastigotes fraction. A statistical analysis showed that the quantity of neutral lipids was significantly higher in *L. am* ^+^ than in both *L. am* ^−^ DLs from the same culture (Figure 4A3, grey histogram), and ctrl DLs (Figure 4A1, white bars) (p<0.01 and p<0.01, figure 4B). In the oleate only-treated sample (Figure 4A2, white histogram), mfi reached 454, proof that the neutral lipids content was higher than in ctrl DLs (Figure 4A1)). This increase was significant (white bars, p<0.04, figure 5B). When exposed to live amastigotes plus oleate (Figure 4A4), DLs contained an even higher neutral lipids content: *L. am* ^+^ DLs gave a higher mfi (figure 4A4, black histogram: 1014) than both *L. am* ^−^ DLs from the same culture (Figure 4A4, grey histogram: 443) and DLs treated by oleate alone (Figure 4A2, white histogram: 454). This increase was significant (p<0.005) in both cases, and also significant when compared to *L. am* ^+^ DLs from the oleate-free sample (black bars, p<0.04, figure 4B). Altogether, these findings indicate that the presence of intracellular amastigotes was associated with increased neutral lipids contents in oleate-treated and untreated DLs. However, since amastigotes have their own LBs, this increased fluorescence could be due to LBs from the host, from the parasite, or from both, and FCM analyses cannot differentiate between them. We therefore conducted apotome analyses of our DL samples by EFM (Figure 5). No LBs were seen in control cultures (image 1), but in all types of DL cultures, i.e. in the presence of *L. am* (image 2), oleate only (image 3) or *L. am* plus oleate (image 4). LBs from oleate-free *L. am* ^+^ DLs were located in the periphery relative to the amastigote nucleus. LBs from oleate-treated *L. am* ^+^ DLs were located similarly or could also be found far from the parasites. LBs differed in size and number depending on culture conditions: they were most numerous in the oleate-treated *L. am* ^+^ DLs, followed by oleate-treated DLs, and oleate-free *L. am* ^+^ DLs. These EFM analyses were unable to determine precisely whether the LBs were of host or parasite origin. To overcome these FCM and EFM limitations, cell cultures were analysed by TEM imaging.

**Figure 6:**
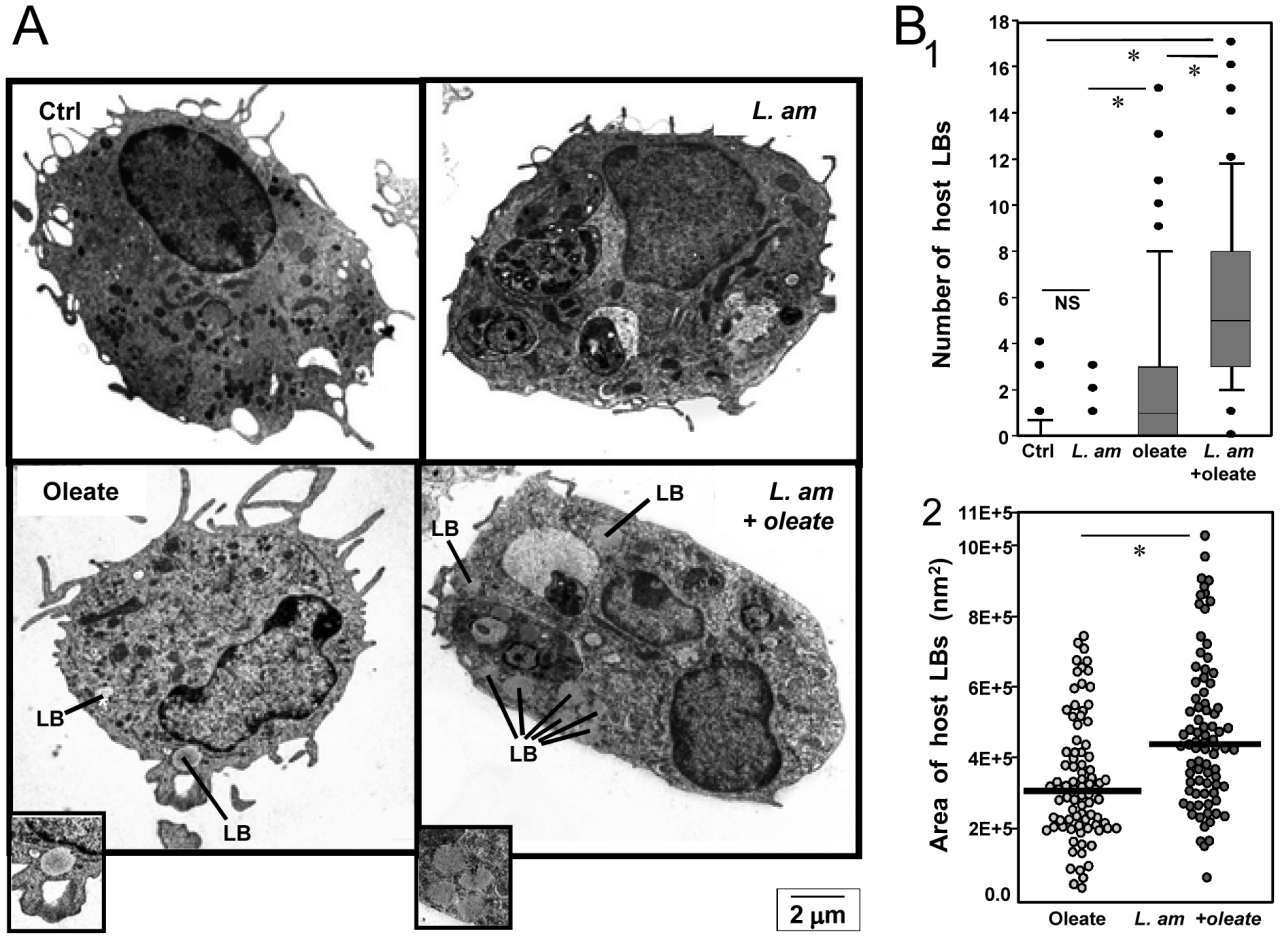
TEM detection and analysis of lipid bodies (LBs) in live *L. am* amastigote-hosting C57BL/6 mouse DLs. **Panel A: TEM pictures of DL cultures exposed or not to live** ***L. am*** **amastigotes and incubated or not with oleate.** Representative pictures are shown for control (Ctrl; upper left panel), oleate-treated (Oleate, lower left panel), amastigote-loaded (*L. am*, upper right panel) and amastigote-loaded treated by oleate (*L. am*+ oleate, lower right panel) C57BL/6 DL cultures. LBs are indicated. **Panel B: Analysis of LBs in control and live** ***L. am*** **amastigote-hosting C57BL/6 DLs incubated or not with oleate.** LB were counted and their area determined in TEM section pictures. The results are represented as box and whisker plots. B1: number of cytosolic LBs in Ctrl DLs and DLs hosting live *L. am* amastigotes in the absence or presence of oleate. B2: LB area in Ctrl DLs and LDs hosting live *L. am* amastigotes in the absence or presence of oleate. Statistical analyses were performed by the Mann Whitney test after analysing at least 60 sections of DL samples. (*): p<0.001.

**Figure 7:**
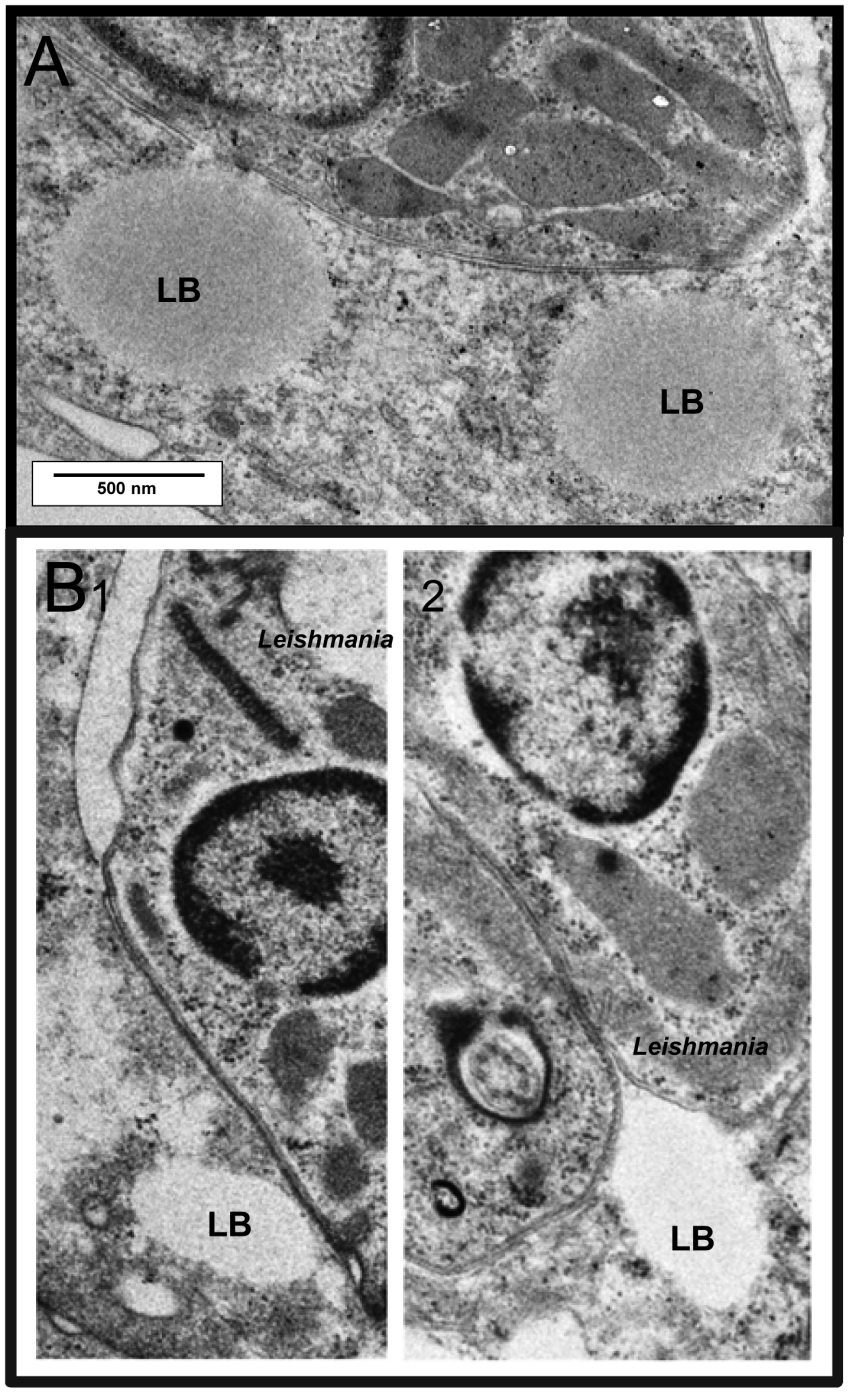
Visualization of contact site with lipid bodies. Live *L. am*-amastigote-hosting DLs, cultured in the presence of oleate, were observed by TEM after conventional (A) or High Pressure Freezing (B) electron microscopy procedures. Scale represents 500 nm.

Osmium tetroxide is highly reactive with unsaturated FA and can therefore be used to reveal the presence of lipid esters derived from the LB core [47]. Since our transcriptomic analysis focused specifically on host cell response, we analysed the number, size and precise location of LBs in the cytoplasm of DLs. These LBs were of low electron density i.e. light grey and uniform in appearance. LBs were observed in only 9.7% of ctrl DLs (Figure 6A and 6B1) and in roughly the same proportion of DLs cultured with *L. am* (6.8%) (Figure 6A and 6B1), which was consistent with the lack of any promotion of *de novo* TAG and cholesterol synthesis suggested by the transcriptomic analysis. Altogether, these findings indicate that the increased BODIPY staining obtained in FCM and EFM analyses of ctrl and *L. am* ^+^ DLs was due to LBs from the parasites, not the host. By contrast, significant numbers of LBs were detected in the cytoplasm of DLs when oleate was added to both *L. am* ^−^ and *L. am* ^+^ samples (Figure 6A, 6B1). LBs were detected at a higher frequency in oleate-treated *L. am* ^+^ DLs (Figure 6A, 6B1). The total number (Figure 6B1) and area (Figure 6B2) of LBs in host cell cytoplasm were significantly higher under this condition. These observations indicate that the reprogramming of lipid metabolism in DLs harbouring *L. am* amastigotes may be phenotypically reflected 24 hours post-infection in the presence of oleic acid. Similar results were obtained for DBA/2 DLs (Figure S4). Interestingly, a high number of the resulting LBs were seen to be in intimate contact with the membrane of PVs (Figure 7A) and parasites (Figure 7B, 7C).

**Figure 8:**
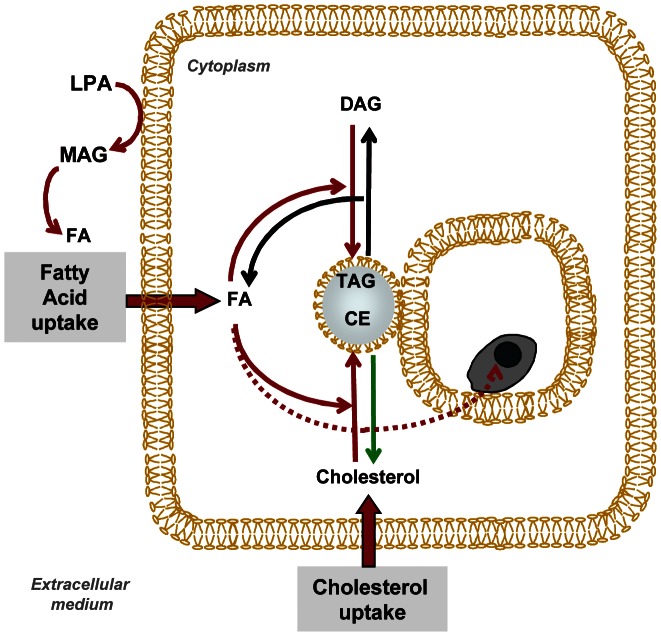
Summary of the subversion of neutral lipid metabolism in C57BL/6 DLs hosting live *L. am* amastigotes. **(i) The increased fatty acid (FA) uptake provides a FA pool that, once esterified, could result in TAG accumulation in lipid bodies. (ii) The increased synthesis of CE through cholesterol import and esterification.** Thin red and green arrows correspond to transcripts that were up- and down-modulated, respectively. Black arrows correspond to un-modulated pathways. Thick red arrows indicate an increased uptake of key lipid constituents necessary for neutral lipid generation and storage.

In conclusion, our study shows that neutral lipid metabolism was rapidly reprogrammed in GM-CSF responsive mouse DLs hosting live *L. am* amastigotes. Whatever the origin - C57BL/6 or DBA/2 mouse - of the bone marrow from which the DLs were generated, this DL reprogramming showed similar features at both the transcriptional and morphological levels, with many LBs being detected in DL cultures to which oleic acid had been added. Parasitized DLs showed coordinated transcriptional modulations that correlated in part to *pparγ* up-regulation and promoted the generation and storage of neutral lipids: TAG and cholesteryl esters. The generation of these lipids was singular since not derived from *de novo* synthesis but from increased import of key constituents, i.e. FAs and cholesterol, from the extracellular milieu, and up-modulation of transcripts involved in their (re-) esterification, such as TAG and CE (Figure 8). When live *L. am* amastigote-hosting DLs were exposed to *oleate*, LBs were located in close proximity to PV, and some established close contacts with the PV membrane. No direct fusion of LB phospholipid monolayer with the PV bilayer membrane was evidenced by the methods used in our study. But in a *Leishmania* we can speculate that LBs store neutral lipids that could be further scavenged by *L. am* amastigotes otherwise shown to be bound to the PV membrane. These LBs could constitute an essential source of both triacylglycerol and cholesterol. As a precursor of major phospholipids such as phosphatidylcholine, phosphatidylethanolamine and phosphatidylserine, TAG from host DL may not only be hydrolysed to provide the live amastigotes with DAG, but could also be important for the synthesis of their key membrane components. CE from host DL may be used to provide the live amastigotes with cholesterol and CE since it is known that the cholesterol present in *Leishmania* parasites is not a product of *de novo* sterol biosynthesis, but is derived from the host (see for review: [48]). Moreover, FAs could be used by amastigotes to produce energy via FA β-oxidation, a process that is known to occur in *Leishmania* and involves several putative enzymes that have been detected by sequencing of the *L. major* genome [49]. β-oxidation is particularly pronounced in amastigotes versus promastigotes, the former relying on FA and amino acids as their main sources of energy [50]. FA like oleate can be involved in the synthesis of polyunsaturated FA (PUFA) through elongases and desaturases, as evidenced in *L. major* (for review: [51]). FA and cholesterol can be used in *L. am* amastigotes for energy and lipid storage, through TAG and CE synthesis, and to generate cytosolic LBs. This *de novo* glycerolipid synthesis involves the activity of several enzymes such as G-3-P acyltransferase (*Lm* GAT) described in *L. major* [52], and GPAT activity expressed in different *Leishmania* species [52], [53].

A recent report has suggested a novel mechanism by which exogenous lipids can affect DC function. Indeed, the increased uptake of FAs- that leads to TAG accumulation in LBs - has been shown to reduce antigen processing and presentation to effector T cells [5]. Interestingly, *in vitro* DLs hosting *L. am* show altered responsiveness to exogenous stimuli, impaired differenciation and migration, and a low capacity to prime naive CD4+T cells [20]. It is not clear how the accumulated lipid could interfere with antigen handling in DCs (for review, [54]). Further investigations should be conducted to determine whether LB-loaded DLs hosting live *L. am* amastigotes can prime and re-activate regulatory T lymphocytes that are reactive to unique peptides delivered from persistent amastigotes. It will be also important to identify the mechanism by which FAs and other lipids might affect DL function and to determine how lipid accumulation relates to and links with membrane, cytosolic and nuclear sites of action of FAs and other lipids on DLs.

Altogether, once loaded with LBs, the DLs hosting live *L. am* amastigotes, may indirectly promote the amastigote-driven remodeling of rodent skin as a dynamic niche where two *L. am* developmental stages durably co- persist: i) those undergoing “controlled” proliferation and those pre-adapted to blood-feeding female sand flies, namely, the next host population upon which *L. am* perpetuation is dependent. Pharmacological agents and transgenic mice are now available to clarify the direct role played by these metabolic active DL organelles in *L. am* replication and/or in the persistence of non-cell-cycling amastigotes.

## Supporting Information

Figure S1 **Gating strategy for the specific sorting of ctrl and** ***Ds*** **Red2−** ***L. am*** **amastigote-hosting DLs. A: Representative FCM profile of the expression of surface MHC II molecules (PE-Cy5 mAb) by DLs present in cultures derived from GM-CSF responsive progenitors present in C57BL/6 mouse bone marrow.** DLs were analysed just before the addition or not of ***Ds*** **Red2−** ***L. am*** amastigotes. Subset 1 did not correspond to DLs and was discarded. Subset 2 expressed surface MHC II molecules and corresponded to DLs. **B: Cell sorting strategy to select and sort ctrl and** ***Ds*** **Red2−** ***L. am*** **amastigote-hosting DLs for Affymetrix-based, genome-wide transcriptional profiles.** A DL control culture (ctrl) and a DL culture placed in contact with live *Ds*Red2− *L. am* amastigotes (LV *L. am*) were analysed 24 hours later without fixation. Representative dot plots of the surface expression of MHC II molecules and the presence of the fluorescence -*Ds*Red2- emitted by the intracellular amastigotes (*Ds*Red2) are shown. The gates were used to select specifically and sort ctrl DLs and live *Ds*Red2 *L. am* amastigote-hosting DLs on the FACSAria cell sorter (see Materials and Methods and [27] for further details).
(TIF)

Figure S2 **Processes associated with an increase in FA uptake in C57BL/6** ***L. am*** **− hosting DLs.** Affymetrix analyses of the modulation of transcripts involved in i) extracellular LPA and MAG hydrolysis, and ii) FA generation, uptake and transport. The fold changes between live *L. am^+^amastigote*-hosting and control DLs are indicated. If the up-modulation of phosphatidic acid phosphatase type - (ppap2b)-coding - *ppab2b*: transcripts correlates with larger quantities of this non-specific phosphatase in plasma membrane lipid rafts and caveolae [55], [56], the latter could act on LPA generating monoacyl glycerol (MAG). It should be noted that if the greater abundance of the lipase-coding *mgl* transcripts correlates with larger quantities of monoglyceride lipase, both at the plasma membrane and intracellularly, it could then prevent MAG, its substrate, from exerting its potent detergent properties on cell membranes. LPA: lysophosphatidic acid, MAG: monoacylglycerol, FA: fatty acids, *ppab2b*: phosphatidic acid phosphatase type 2B, *mgl*: monoglyceride lipase, *cav1*: caveolin-1, *cav2*: caveolin-2, *fabp4*: fatty acid binding protein 4, *fabp5*: fatty acid binding protein 5, *slc27a1*: solute carrier family 27, member a1.
(TIF)

Figure S3 **FCM analysis of BODIPY FL-C16 uptake in control DLs and DLs hosting** ***Ds*** **Red2** ***L. am*** **amastigotes (A) and epifluorescence microscopy (B).** **Panel A**: *Ds*Red2− *L. am* amastigotes were added or not to DL cultures at a ratio of 5∶1. Twenty four hours later, fluorescent palmitic acid (BODIPY FL C16, green fluorescence), was added to control (Ctrl, upper panel) and *L. am* DL cultures (*L. am*, lower panel) for 30 minutes at 34°C. The DLs were stained with anti- MHC II mAbs conjugated to PE-Cy5. Samples were analysed without fixation. After specific gating on MHC II^+^ DLs, the analysis was performed on bi-parametric dot plots showing *Ds*Red2 and BODIPY FL C16 fluorescence signals. Mean green fluorescence (related to palmitic acid) values are indicated for control (ctrl) and amastigote-loaded (*L.am*) DLs where black and red gates correspond to amastigote-free (*Ds*Red2^−^) and live *L. am* amastigote-hosting (*Ds*Red2^+^) DLs respectively. **Panel B: Epifluorescence analysis of BODIPY FL-C16 uptake in control DLs and DLs hosting** ***Ds*** **Red2** ***L. am*** **amastigotes.** DLs were gently deposited on separate coverslips before being fixed with paraformaldehyde and examined under a Zeiss microscope fitted with an ApoTome module. DL and amastigote nuclei were stained with Hoechst 33,342 (blue spots indicated by arrow heads). Note the presence of BODIPY FL C16 in the cytoplasm of both DLs and amastigotes located in single PVs. The upper amastigote is zoomed in the additional insert.
(TIF)

Figure S4 **TEM detection of lipid bodies (LBs) in DBA/2 DL cultures.** **Panel A: TEM pictures of DBA/2 DL cultures exposed or not to live** ***L. am*** **amastigotes and incubated or not with oleate.** Representative pictures are shown for control (Ctrl; upper left panel), oleate-treated (Oleate, lower left panel), amastigote-loaded (*L. am*, upper right panel) and amastigote-loaded treated by oleate (*L. am*+ oleate, lower right panel) DBA/2 DL cultures. LBs are indicated. **Panel B: Analysis of LBs in control and** ***l. am*** **-hosting DBA/2 DLs.** The number of cytosolic LBs in DLs hosting live *L. am* amastigotes was determined in the absence or presence of oleate (B1). LBs were counted in TEM section pictures and the results are represented as box and whisker plots. LB areas in DLs incubated with oleate and loaded or not with amastigotes are represented in (B2). Statistical analysis was performed by the Mann Whitney test after analysing at least 60 sections of DL samples. (*): p<0.001.
(TIF)
