# Supplementary figures and images for: Reprogramming Neutral Lipid Metabolism in Mouse Dendritic Leucocytes Hosting Live Leishmania amazonensis Amastigotes
Source: PLoS Negl Trop Dis. 2013 Jun 13;7(6):e2276. doi: 10.1371/journal.pntd.0002276 (PMC3681733; doi:10.1371/journal.pntd.0002276)

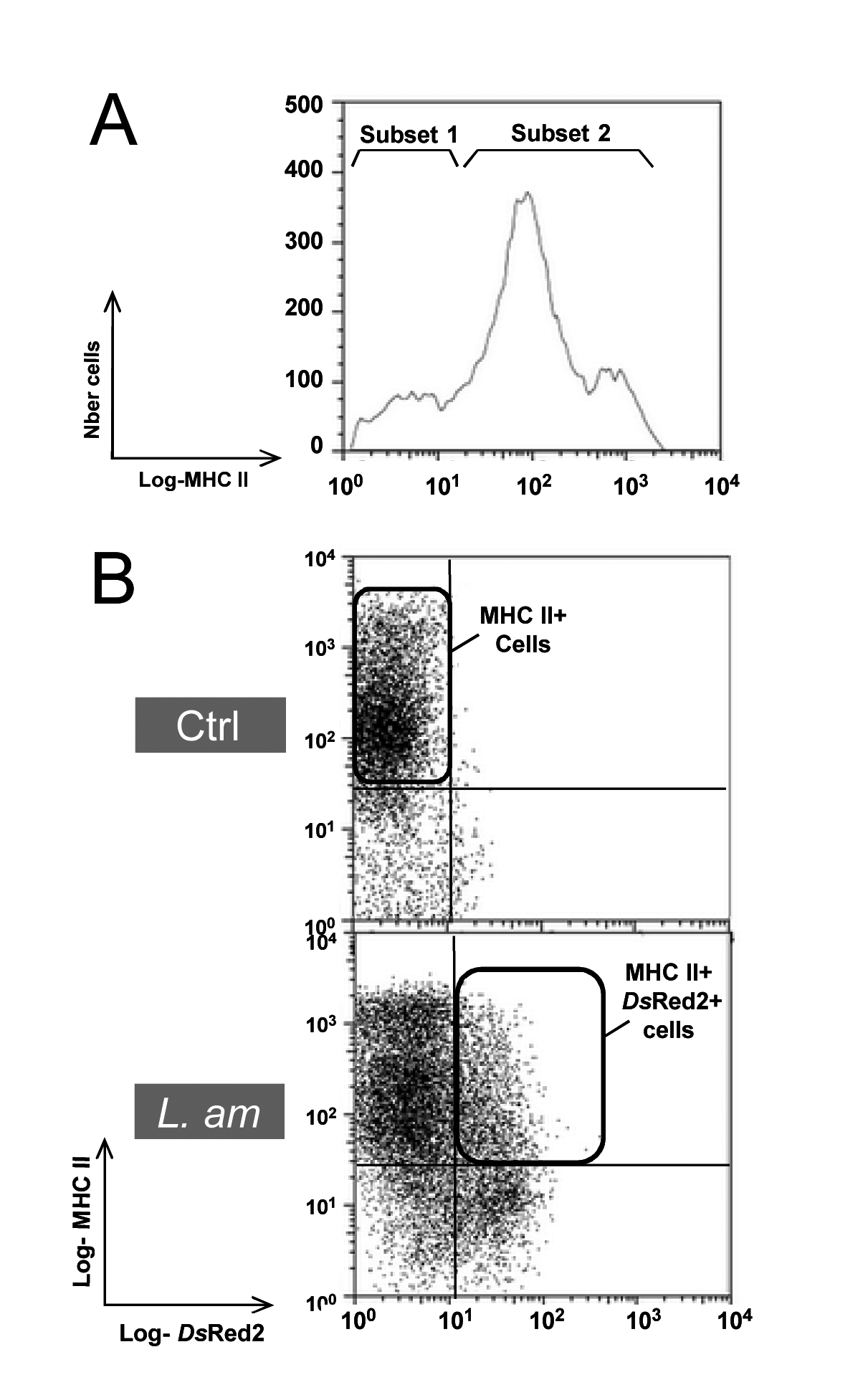

Supplement: Figure S1 — Gating strategy for the specific sorting of ctrl and Ds Red2− L. am amastigote-hosting DLs. A: Representative FCM profile of the expression of surface MHC II molecules (PE-Cy5 mAb) by DLs present in cultures derived from GM-CSF responsive progenitors present in C57BL/6 mouse bone marrow. DLs were analysed just before the addition or not of Ds Red2− L. am amastigotes. Subset 1 did not correspond to DLs and was discarded. Subset 2 expressed surface MHC II molecules and corresponded to DLs. B: Cell sorting strategy to select and sort ctrl and Ds Red2− L. am amastigote-hosting DLs for Affymetrix-based, genome-wide transcriptional profiles. A DL control culture (ctrl) and a DL culture placed in contact with live DsRed2− L. am amastigotes (LV L. am) were analysed 24 hours later without fixation. Representative dot plots of the surface expression of MHC II molecules and the presence of the fluorescence -DsRed2- emitted by the intracellular amastigotes (DsRed2) are shown. The gates were used to select specifically and sort ctrl DLs and live DsRed2 L. am amastigote-hosting DLs on the FACSAria cell sorter (see Materials and Methods and [27] for further details). (TIF) [file pntd.0002276.s001.tif]

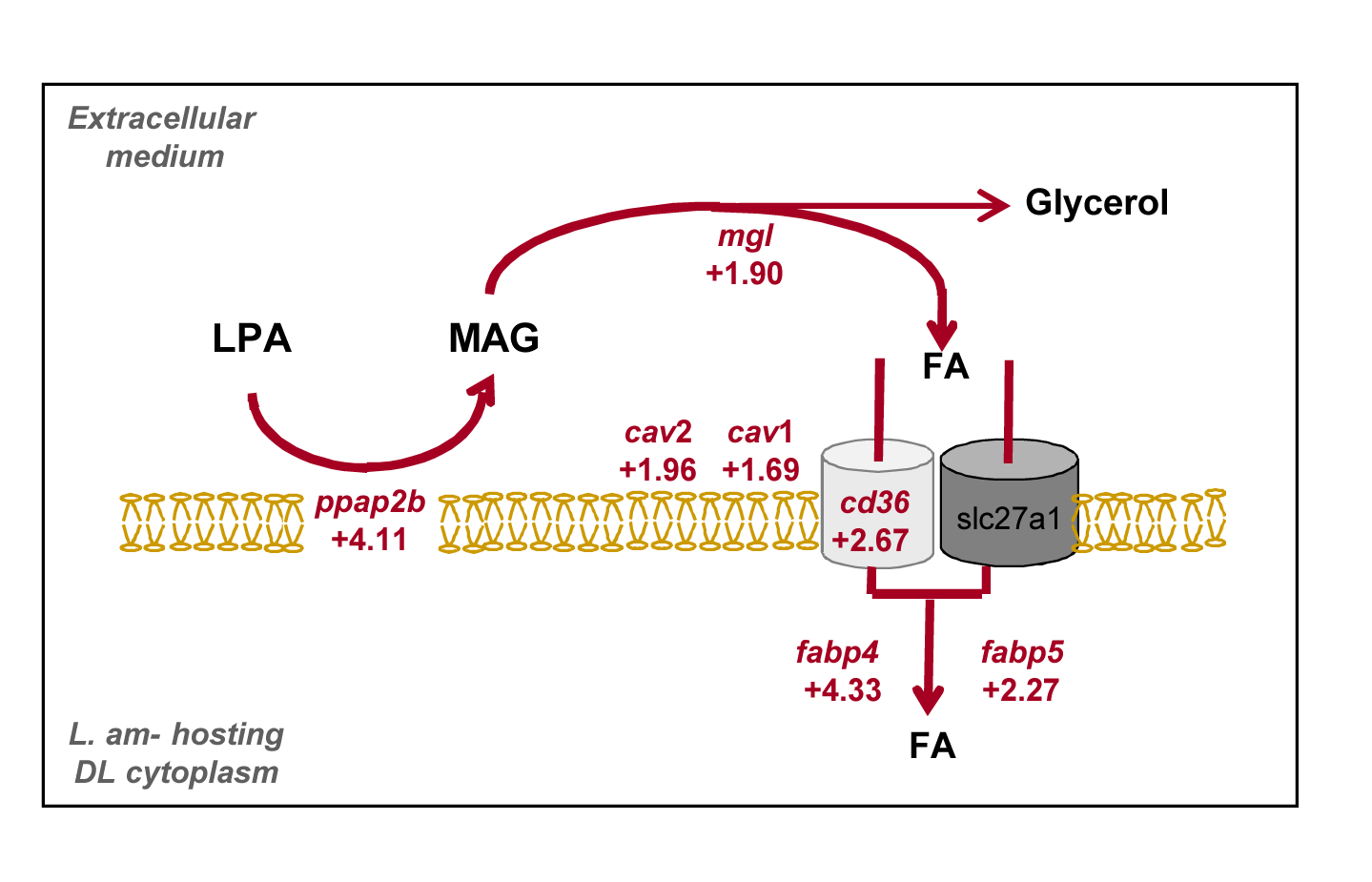

Supplement: Figure S2 — Processes associated with an increase in FA uptake in C57BL/6 L. am − hosting DLs. Affymetrix analyses of the modulation of transcripts involved in i) extracellular LPA and MAG hydrolysis, and ii) FA generation, uptake and transport. The fold changes between live L. am+amastigote-hosting and control DLs are indicated. If the up-modulation of phosphatidic acid phosphatase type - (ppap2b)-coding - ppab2b: transcripts correlates with larger quantities of this non-specific phosphatase in plasma membrane lipid rafts and caveolae [55], [56], the latter could act on LPA generating monoacyl glycerol (MAG). It should be noted that if the greater abundance of the lipase-coding mgl transcripts correlates with larger quantities of monoglyceride lipase, both at the plasma membrane and intracellularly, it could then prevent MAG, its substrate, from exerting its potent detergent properties on cell membranes. LPA: lysophosphatidic acid, MAG: monoacylglycerol, FA: fatty acids, ppab2b: phosphatidic acid phosphatase type 2B, mgl: monoglyceride lipase, cav1: caveolin-1, cav2: caveolin-2, fabp4: fatty acid binding protein 4, fabp5: fatty acid binding protein 5, slc27a1: solute carrier family 27, member a1. (TIF) [file pntd.0002276.s002.tif]

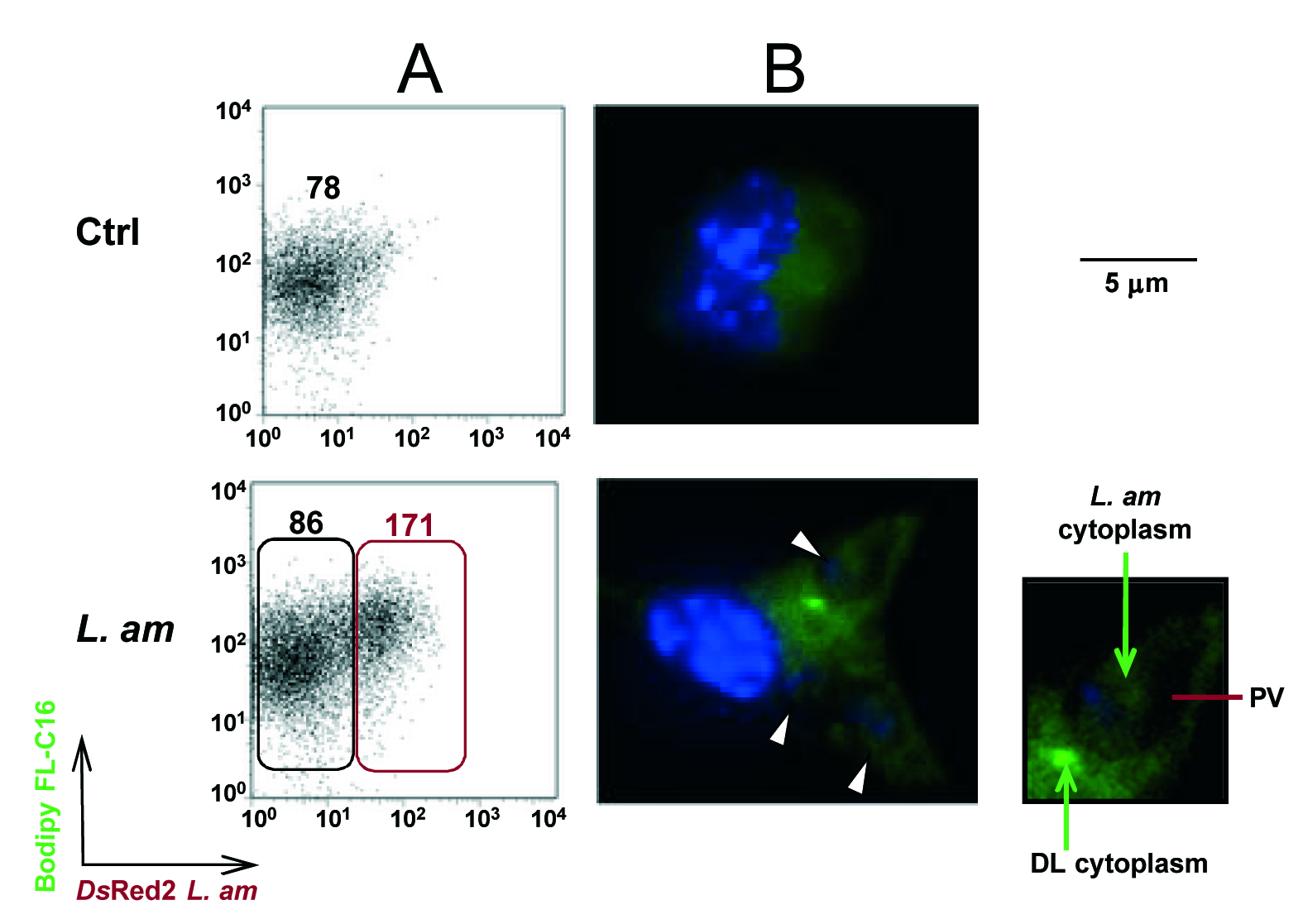

Supplement: Figure S3 — FCM analysis of BODIPY FL-C16 uptake in control DLs and DLs hosting Ds Red2 L. am amastigotes (A) and epifluorescence microscopy (B). Panel A: DsRed2− L. am amastigotes were added or not to DL cultures at a ratio of 5∶1. Twenty four hours later, fluorescent palmitic acid (BODIPY FL C16, green fluorescence), was added to control (Ctrl, upper panel) and L. am DL cultures (L. am, lower panel) for 30 minutes at 34°C. The DLs were stained with anti- MHC II mAbs conjugated to PE-Cy5. Samples were analysed without fixation. After specific gating on MHC II+ DLs, the analysis was performed on bi-parametric dot plots showing DsRed2 and BODIPY FL C16 fluorescence signals. Mean green fluorescence (related to palmitic acid) values are indicated for control (ctrl) and amastigote-loaded (L.am) DLs where black and red gates correspond to amastigote-free (DsRed2−) and live L. am amastigote-hosting (DsRed2+) DLs respectively. Panel B: Epifluorescence analysis of BODIPY FL-C16 uptake in control DLs and DLs hosting Ds Red2 L. am amastigotes. DLs were gently deposited on separate coverslips before being fixed with paraformaldehyde and examined under a Zeiss microscope fitted with an ApoTome module. DL and amastigote nuclei were stained with Hoechst 33,342 (blue spots indicated by arrow heads). Note the presence of BODIPY FL C16 in the cytoplasm of both DLs and amastigotes located in single PVs. The upper amastigote is zoomed in the additional insert. (TIF) [file pntd.0002276.s003.tif]

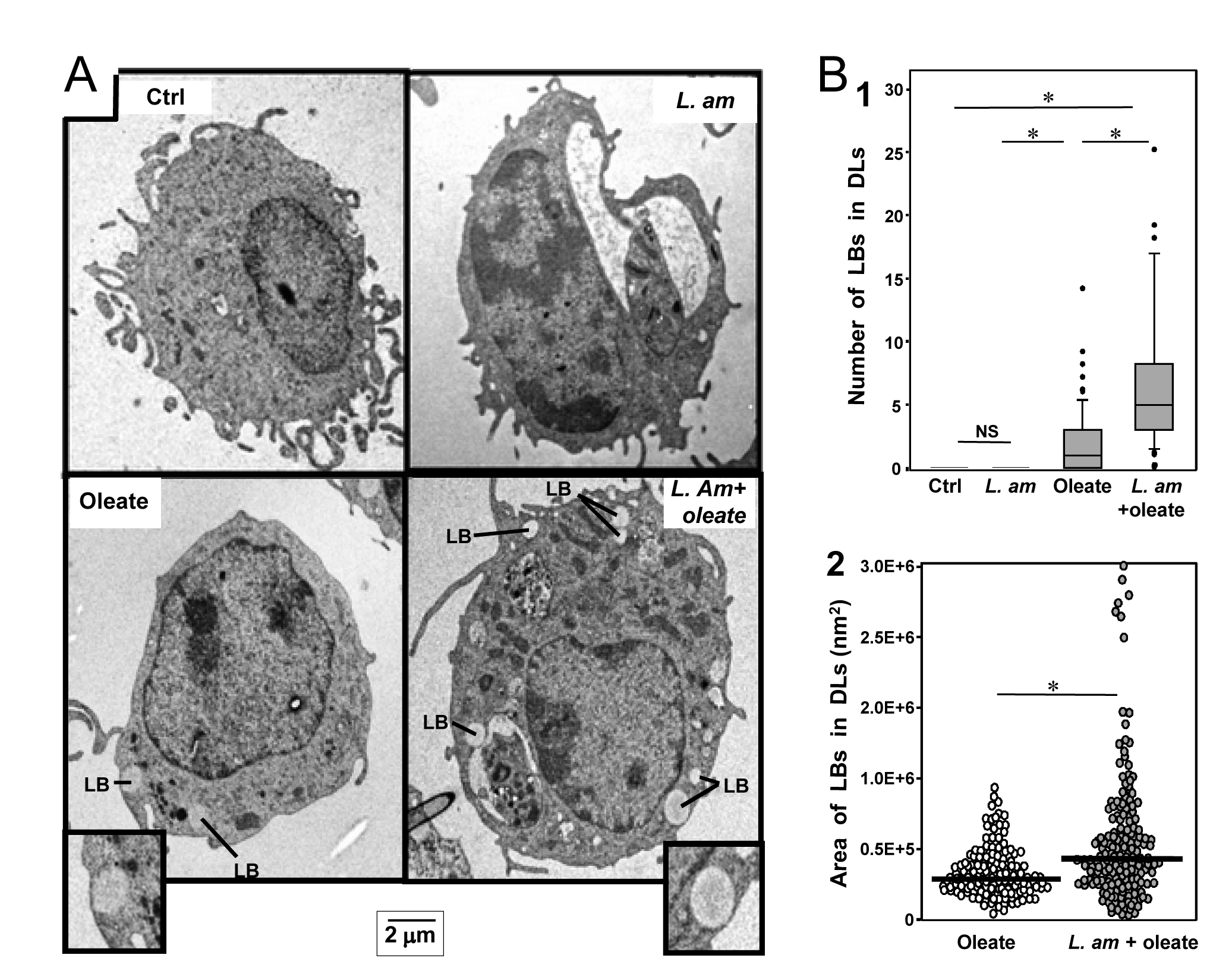

Supplement: Figure S4 — TEM detection of lipid bodies (LBs) in DBA/2 DL cultures. Panel A: TEM pictures of DBA/2 DL cultures exposed or not to live L. am amastigotes and incubated or not with oleate. Representative pictures are shown for control (Ctrl; upper left panel), oleate-treated (Oleate, lower left panel), amastigote-loaded (L. am, upper right panel) and amastigote-loaded treated by oleate (L. am+ oleate, lower right panel) DBA/2 DL cultures. LBs are indicated. Panel B: Analysis of LBs in control and l. am -hosting DBA/2 DLs. The number of cytosolic LBs in DLs hosting live L. am amastigotes was determined in the absence or presence of oleate (B1). LBs were counted in TEM section pictures and the results are represented as box and whisker plots. LB areas in DLs incubated with oleate and loaded or not with amastigotes are represented in (B2). Statistical analysis was performed by the Mann Whitney test after analysing at least 60 sections of DL samples. (*): p<0.001. (TIF) [file pntd.0002276.s004.tif]
